# The Association Between Academic Achievement and Subsequent Youth Offending: A Systematic Review and Meta-Analysis

**DOI:** 10.1007/s40865-025-00266-9

**Published:** 2025-03-20

**Authors:** M. Lankester, C. Coles, A. Trotter, S. Scott, J. Downs, H. Dickson, A. Wickersham

**Affiliations:** 1 King’s College London, Institute of Psychiatry, Psychology & Neuroscience, 16 De Crespigny Park, London, SE5 8AF UK; 2 King’s College London, Department of Forensic and Neurodevelopmental Science, Institute of Psychiatry, Psychology & Neuroscience, 16 De Crespigny Park, London, SE5 8AF UK; 3 South London and Maudsley NHS Foundation Trust, 16 De Crespigny Park, London, SE5 8AF UK; 4 King’s College London, CAMHS Digital Lab, Department of Child and Adolescent Psychiatry, Institute of Psychiatry, Psychology & Neuroscience, 16 De Crespigny Park, London, SE5 8AF UK

**Keywords:** Academic achievement, Youth offending, Delinquency, Systematic review, Meta-analysis

## Abstract

The association between academic achievement and youth offending has yet to be clearly quantified. This meta-analysis aims to provide a robust estimate of the longitudinal association between academic achievement and subsequent youth offending. We searched PsycINFO, ERIC, British Education Index, and Web of Science from inception to 2 April 2024 using a comprehensive search strategy. We identified eligible studies reporting on the association between academic achievement and subsequent youth offending, as measured using self-report or administrative records up to the age of 25 years. Correlation coefficients and odds ratios were pooled in a meta-analysis. Effect modifiers were investigated in a sub-group analysis, and other findings were narratively synthesised. PROSPERO record: CRD42023402103. Seventeen studies were included, of which eight were pooled in a meta-analysis. The findings showed a small but statistically significant association between lower academic achievement and youth offending (pooled Fisher *z* =  − 0.21, 95% CI [− 0.29, − 0.12], *I*^2^ = 98.4%). Sub-group analysis and narrative synthesis yielded mixed findings on the possible role of measurement timing and offence types. Lower academic achievement was associated with subsequent youth offending, underlining the need for a child-first approach to early prevention and intervention strategies in educational and forensic settings.

**Supplementary Information:**

The online version contains supplementary material available at 10.1007/s40865-025-00266-9.

## Introduction

In England and Wales, the year ending March 2023 saw a 9% increase in arrests of children and an 8% increase in sentencing occasions involving children compared to the previous year (Youth Justice Board for England & Wales, 2024). There are substantial costs associated with youth offending, encompassing physical and mental health services, law enforcement, youth justice, and custody services (Hemphill et al., 2015). In the UK alone, reports reveal an expenditure of £158 m on youth custody services and £94 m on youth justice board services from 2020 to 2021 (National Audit Office, 2023). The social and economic cost of youth crime highlights the need for initiatives that prioritise early identification and protection of children against future involvement with the criminal justice system.

It has long been recognised that educational experiences contribute to adolescents’ risk of engagement in criminal activities (Department for Education & Ministry of Justice, 2023; Rutter et al., 1979). One factor frequently explored within this context is the role of academic achievement (Farrington, 2003). Longitudinal studies have shown low academic achievement as a robust predictor of future criminal behaviour (Martins et al., 2022). This is in keeping with the integrated cognitive antisocial potential (ICAP) theory (Farrington, 2020). This theory attempts to explain the mechanisms underlying ‘antisocial potential’, a construct that represents an individual’s potential to engage in antisocial behaviour, and how this potentially translates to criminal offending. In this context, ‘school failure’ (alongside low income and unemployment) is proposed as a long-term contributor to antisocial potential. This is because individuals may turn to antisocial behaviour to satisfy their goals and motivations, both in terms of status and financial stability, if they are struggling to meet their needs through legitimate means (Farrington, 2020). Low academic achievement has been theorised to weaken social bonds and adherence to societal norms, impede the development of self-efficacy and positive sense of self, and limit economic opportunities (Donnellan et al., 2005; Farrington, 2003). Furthermore, low academic achievement leads to greater associations with delinquent peers and groups, heightening short-term AP by increasing the situational pressures on an individual to offend (Farrington, 2018).

An advantage of concentrating on academic achievement is that it represents a modifiable risk factor that could potentially be targeted early in childhood and adolescence. If high academic achievement is associated with reduced offending or delinquency outcomes, then there is significant motivation to establish whether it has a causal role and develop school-based programmes aimed at improving educational outcomes. Understanding associations between academic achievement and violent behaviour or aggression was the focus of a meta-analysis by Savage and colleagues (Savage et al., 2017). This meta-analysis reported that academic achievement may be a stronger predictor of violent criminal behaviour than non-violent. Their findings support the differential aetiology of violence hypothesis (Savage & Wozniak, 2016), which posits that violent behaviour should be distinguished from other forms of criminal behaviour. Savage et al. (2017) propose that poor academic achievement may be a specific predictor of violent offending due to the negative association between intelligence and violence. Other theories suggest that school problems lead to frustration and anger which can manifest as aggression (Eme, 2020).

However, aspects of Savage et al.’s (2017) study design limit our ability to generalise these findings to the association between academic achievement and youth offending more generally. First, their eligibility criteria excluded studies of ‘delinquency’ as a general construct, because they wanted to be able to differentiate between associations specifically for violent versus non-violent offending. To fully capture associations between academic achievement and offending outcomes among young people, it would be important to capture studies of delinquency, this being a widely used term in the literature to describe law-breaking behaviour among juveniles. Second, Savage et al. (2017) included studies which measured physical aggression in general (such as intention to cause harm); while criminal offending is a potential outcome of aggression, they represent distinct constructs (Berlin et al., 2021), warranting a further investigation focusing on offending. Additionally, Savage et al. (2017) included cross-sectional studies. The current study only includes longitudinal studies where attainment is investigated prior to offending, allowing investigation of the temporal order of events that creates potential for making causal inferences. We exclusively focus on the impact of academic achievement, excluding studies investigating other education-related outcomes such as general intelligence. Our operationalisation of academic achievement is important as it measures a potentially modifiable outcome, students’ grades and scores, which determines further academic opportunities.

The aim of the current meta-analysis was to investigate the association between academic achievement and subsequent youth offending. We hypothesised that there is an association between lower academic achievement and increased subsequent youth offending. Our secondary aims were to investigate whether this association varied according to offence type or by timing of achievement and offending measurements.

## Methods

This meta-analysis followed the Preferred Reporting Items for Systematic Reviews and Meta-Analyses (PRISMA) guidelines (Table S1) (Page et al., 2021). The protocol can be viewed on PROSPERO at https://www.crd.york.ac.uk/prospero/display_record.php?RecordID=402103.

### Eligibility Criteria

Study eligibility criteria are summarised in Table 1, with more detail provided on each criterion below.

**Table 1:** Study eligibility criteria

Parameter	Inclusion criteria	Exclusion criteria
Population	- Participants aged up to 25 years old at the time of outcome measurement	- No restrictions
Exposure	- Educational achievement as measured using self- or informant-reports, or administrative records	- School dropout, general intelligence, aptitude, or ability only
Comparison	- No restrictions	- No restrictions
Outcome	- Youth offending or delinquency as measured using self- or informant-reports, or administrative criminal records	- Externalising problems or conduct disorder only
Study design	- Investigate and report on the association between educational achievement and subsequent youth offending and delinquency - Publications in English with full text available - Primary research published in a peer-reviewed journal - Quantitative longitudinal studies with prospective data collection	- Aim to conduct or evaluate an intervention during the observed study period

#### Population

To capture youth offending and delinquency, we focused on offending measured up to age 25 years. This represents a significant period of biological and social change before fully mature adulthood (Sawyer et al., 2018). Furthermore, due to variations in the age at which compulsory education ends in different countries, this ensured a sufficient follow-up period for the offending outcome to occur.

#### Exposure and Outcome Variables

Eligible studies reported on the relationship between academic achievement (exposure) and subsequent offending and delinquency (outcome). We included any measure of academic achievement (self- or informant-report or from administrative records) including, but not limited to, grades, grade point average (GPA), and test scores, in recognition that, internationally, different education systems measure academic achievement differently. However, school dropout, general intelligence, aptitude, or ability-only measures were excluded as constructs, which can be associated with, but are distinct from, academic achievement. Youth offending or delinquency could likewise be self- or informant-reported or derived from administrative records, and externalising problems and conduct disorder (CD) were excluded as distinct constructs.

#### Study Design

Included studies were quantitative, prospective longitudinal studies, to help infer a direction of effect. As a result, cross-sectional studies, case reports, clinical vignettes, exclusively qualitative studies, and retrospective studies were excluded. Studies were also original research reported in full, such that reviews, meta-analyses, commentaries, letters to the editor, opinion pieces, and editorials were also excluded. Furthermore, studies that aimed to conduct or evaluate an intervention during the study period were excluded, such as randomised controlled trials, as this might influence the observed associations. Due to limited resources for translation and time constraints, included studies were English and published in peer-reviewed journals. This excluded ‘grey literature’, such as books, reports, and theses. Finally, the full text had to be available, including reported associations between academic achievement and youth offending.

### Search Strategy

PsycINFO, Education Resources Information Centre (ERIC), British Education Index (BEI), and Web of Science were searched on 26 October 2021. No date range restrictions were applied to the search, but English language limits were used. The searches included keyword combinations capturing the underlying concepts of (1) academic achievement, (2) offending, and (3) youth. We additionally included subject headings in the searches, which were adapted to each database’s thesaurus. Full search strategies for each database can be found in Supplement 1. A search update was performed on 2 April 2024.

### Data Management and Selection Process

De-duplication was conducted in Endnote. Eligibility screening then took place in two stages: title and abstract screening, followed by full-text screening. For the original search, co-author ML conducted screening, and co-author CC conducted independent second screening for 10% at each stage. Researcher agreement was 97% following independent title and abstract screening and 90% following independent full-text screening. Disagreements were adjudicated by co-author AW. For the search update, screening was conducted by CC.

### Data Extraction

Data extracted from eligible studies included first author, publication year, country of study, sample characteristics (number of participants, ethnicity, gender, age and grade at the time of exposure and outcome measurement, any additional participant inclusion or exclusion criteria), exposure and outcome variable measurement, follow-up period, covariates adjusted for, and relevant findings. Any studies, which drew from the same longitudinal data sources, were recorded separately in data extraction due to the potential for providing insights into different covariates and effect modifiers. For the original search, ML conducted data extraction, and CC conducted independent second data extraction for 10%. Disagreements were adjudicated by AW. For the search update, CC and AW conducted data extraction.

### Quality Assessment

Risk of bias was assessed using a modified 11-item version of the Newcastle–Ottawa Scale (NOS) for cohort studies (Wells et al., n.d.; Wickersham et al., 2021), which assesses participant selection, comparability, and outcomes (Supplement 2). Items were tailored to reflect the focus of this meta-analysis on academic achievement and youth offending, such as using administrative records compared to self or informant-report for exposure and outcomes measurements. Risk of bias was scored out of a total of 13 points (higher scores indicate better study quality). Scores of 10–13, 6–9, and 0–5 indicate low, moderate, and high risk of bias, respectively (Epstein et al., 2020). For the original search, ML conducted quality assessment, and CC conducted independent quality assessment on 10%, with disagreements adjudicated by AW. Researcher agreement was 85%. For the search update, CC conducted quality assessment.

### Data Synthesis and Analysis

Correlation coefficients were the most frequently reported effect sizes among the included studies and were, therefore, pooled in a meta-analysis. The second most frequently reported effect size was odds ratios (OR), which we transformed into correlation coefficients and also included in the meta-analysis (Borenstein et al., 2009). If studies reported relevant effect sizes for multiple timepoints, we took the effect size for the two most distal timepoints to investigate longitudinal effects. If studies still reported multiple relevant effect sizes for separate or overlapping subgroups, we took the mean average correlation coefficient (taken by averaging their Fisher’s *z* transformations) and the total sample size. However, where it was not possible to estimate a total sample size for combined groups, we took the effect size for the largest subgroup instead. Any correlation coefficients that reported on the reverse association (i.e. academic underachievement and offending) were multiplied by − 1 to ensure all associations included in the meta-analysis were investigating the same direction. Correlation coefficients underwent Fisher’s *z* transformations and were pooled in a random effects (restricted maximum likelihood estimator) meta-analysis in R (version 4.1.2) using the ‘rma’ function in the ‘metafor’ package (Viechtbauer, 2010). Heterogeneity was investigated using Cochran’s *Q* and *I*^2^ statistics (Higgins et al., 2003), and publication bias was assessed using Egger’s test and a contour-enhanced funnel plot (Egger et al., 1997).

We conducted subgroup analyses to assess whether using administrative records versus self- or informant-reports for exposure and outcome measurements impacted the observed association. We also conducted a subgroup analysis to assess whether the timing of academic achievement and youth offending measurements impacted observed associations. We categorised studies into two groups based on follow-up duration, using the mean follow-up period of 6 years across all eligible studies from the original search. Hence, studies with a follow-up of less than 6 years versus 6 years or greater were allocated to the shorter and longer follow-up subgroups, respectively. If studies included multiple follow-up periods, they were categorised according to the longest follow-up period. For these subgroup analyses, we first obtained the estimated average effects of the two subgroups using separate random-effect models. Then, using a fixed-effect model, we compared the two estimates (Rubio-Aparicio et al., 2017; Viechtbauer, 2010).

A narrative synthesis was conducted to summarise studies that could not be included in the meta-analysis and to further explore our secondary research questions (as there were not sufficient subgroups to investigate the potentially modifying role of offence type in the meta-analysis).

## Results

### Summary of Study Characteristics

**Fig. 1:**
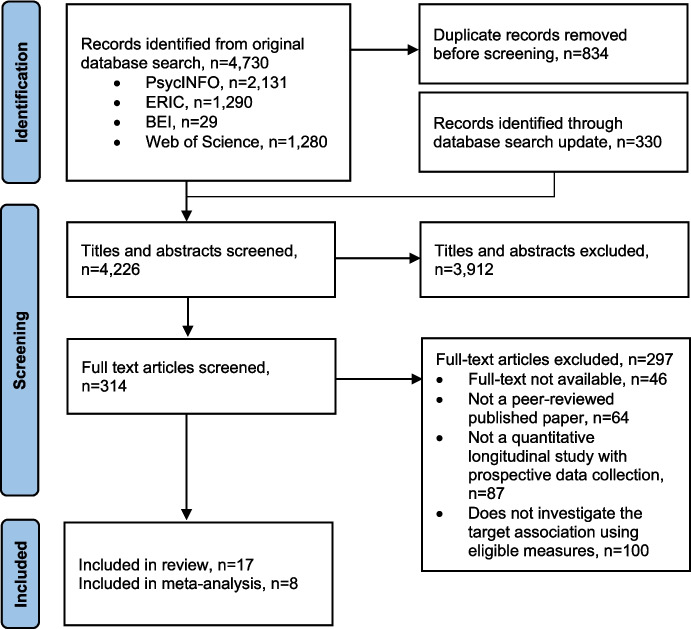
PRISMA flow diagram

Of the original 4730 records identified from database searches, and the additional 330 records identified in the search update, 17 studies were eligible for inclusion (Fig. 1, Tables 2 and Table S2). The included studies had publication years ranging from 2000 to 2022 and sample sizes ranging from *n* = 389 to *n* = 451,054.

**Table 2:** Study characteristics

Citation	Country of study	*n*	Ethnicity	Gender balance (% male)	Age and grade at academic achievement measurement	Academic achievement measurement	Age and grade at youth offending measurement	Youth offending measurement	Follow-up period	Total risk of bias score (max = 11)
(Choi, 2007)	USA	13,377	At wave 1: 6.3% = API Asian Pacific Islander American; *n* = 843), 19.9% is black (*n* = 2663), 17.2% Hispanic (*n* = 2298), 52.7% white (*n* = 7051), and 3.9% multiracial (*n* = 522)	48.8%*	M = 16.09; SD = 1.76; range = 12–18 years old*; 7th–12th grade	Self-reported grades in English or Language Arts, Mathematics, History or Social Sciences, and Science. GPA was calculated by averaging four items of the grades (continuous)	Range = 13–19 years old*	Self-reported youth offending behaviour: aggressive and non-aggressive delinquent offences (binary)	1 y*	6
(Crosnoe, 2002)	USA	2899	Divided according to curriculum track: College prep: White (65.6%), African American (7.8%), Hispanic (8.0%), Asian (18.4%) Non-college prep: White (62.7%), African American (6.6%), Hispanic (16.8%), Asian (13.9%)	College prep: 42.7%; Non-college prep: 46.1%	College prep: M = 15.10; SD = 0.90; Non-college prep: M = 15.01; SD = 0.99	Self-reported GPA (continuous)	At wave 2 (year 2): 15–16 years old*; 10th grade* At wave 3 (year 3): 16–17 years old*; 11th grade*	Delinquency as measured using self-reported frequency of engagement with 6 delinquent behaviour items (categorical)	1–2 y*	7
(Defoe et al., 2013)	USA	445	At wave 1: 57% African American. Other Ethnicity data were not provided	100%	Range = 11–14 years old; 5th–10th grade*	Mean ratings by child, parents, and teachers on reading, mathematics, writing, and spelling. Additionally, parent and self-ratings of up to three other subjects (continuous)	Range = 12–15 years old; 6th–10th grade*	Youth offending as measured using self-reported frequency of committing a crime (25 types) in the previous year (continuous)	1–4 y*	5
(Dodge et al., 2008)^†^	USA	754	43% African American. Other Ethnicity data were not provided	50%	Range = 6/7–10 years old* 1st–4th grade	Grade retention and core subject failing were scored from administrative school records (binary)	Range = 15–17 years old*; 10th and 11th grade	Delinquency as measured using 32 items taken from existing self- and parent-report delinquency measures was summed as an index of adolescent violent behaviour (binary)	9–11 y*	5
(Farrington et al., 2016)^†^	UK	411	Not reported	100%	Range = 8–10 years old; year 4–6*	Administrative school records of English, arithmetic and verbal reasoning tests (categorical)	Range = 10–18 years old; year 6–13*	Youth offending as measured using conviction records (binary)	< 1–10 y*	9
(Felson & Staff, 2006)^†^	USA	10,439	75.5% white; 11.4% black; 8.6% Hispanic; 3.5% Asian, and 1.0% American Indian	47.6%	Range = 15–16 years old*; 10th grade	Official transcript grades, GPA for all 9th and 10th-grade classes, and 2 averaged standardised tests on reading comprehension and mathematics (continuous)	Range = 17–18 years old*; 12th grade	Self-reported frequency across four items assessing delinquency behaviour outside the classroom (continuous)	2 y*	8
(Gremmen et al., 2019)^†^	The Netherlands	1219	Not reported	At wave 1: 49.3%	M = 13.69; range = 12–15 years old*; 7th, 8th, 9th grade	GPA from administrative data: school report card grades (continuous)	Range = 12–16 years old*; 7th, 8th, and 9th grade	Delinquency as measured using self-reported frequency of 18 types of antisocial behaviour (continuous)	< 1 y*	8
(Hemphill et al., 2016)^†^	Australia	437	Not reported	45.1%	Grade 5: M = 11.0; SD = 0.41; Grade 9: M = 17.0; SD = 0.4	Self-reported questionnaire of grades (binary)	Range = 18–19	Violent offending as measured using three self-report questions of violent offending behaviour (binary)	8 y	7
(Karriker-Jaffe et al., 2021)^†^	Sweden	451,054	Not reported	100%	End of Grade 9: range = 15–16 years* (usually 16)	Average educational attainment from administrative data; standardised to M = 0, SD = 1 (continuous)	Range = 19–25 years	Convictions in lower courts from the Swedish Crime register (binary)	3–9 y*	10
(Lee, 2013)^†^	South Korea	2866*	Not reported	50%	M = 13.2; SD = 0.4; 8th,9th, 10th grade	Self-reported achievement in Korean Language (mother tongue), English, and Mathematics (continuous)	Range = 14–17 years old*; 9th, 10th, and 11th grade	Delinquency as measured using self-report of 14 items of delinquent behaviours from the KYPS (continuous)	4 y	7
(Lesner et al., 2022)	Denmark	93,859	Not reported	48.9%	Grade 6: range = 11–12 years old* Grade 8: range = 13–14 years old*	National standardised average test scores on reading and maths (continuous)	Year following grade 9, > 16 years old*	Official records of charges, convictions and police contacts for non-traffic-related offences (binary)	2–5 y*	10
(Mercer et al., 2016)	UK	389	At wave 1: 87% Caucasian. Other ethnicity data were not provided	100%	Range = 8–10 years old; year 4–6*	Parent/child/teacher/peer questionnaires and interviews of primary school achievement (continuous)	Median (wave 2) = 14.9 years old; median (wave 3) = 18.7 years old	Self-reported engagement in delinquent offending; conviction records (both self-report and conviction records were recorded separately; binary)	6–10 y*	6
(Sabates, 2008)	UK	Not reported	Not reported	Not reported	M = 16 years old; end of secondary schooling (year 11*)	Official records from the Local Education Authority School Information System (LEASIS)—five or more GCSEs at grades A–C (binary)	17–22 years old	Youth offending as measured using official conviction data obtained from the Offenders Index (OI) database (continuous)	1–6 y*	4
(Savage & Ellis, 2019)	USA	4348	Not reported	46.5%	Range = 12–18 years old*; 7th–12th grade	GPA was estimated using self-reported grades in maths, English/language arts, history/ social studies, and science (continuous)	M = 15.8 years old; range = 13–18 years old; 8th–12th grade*	Youth offending as measured using self-reported frequency of violent and non-violent offending (continuous)	1 y	7
(Savolainen et al., 2012)	Finland	4645	Not reported	47%	M = 15; range = 14–15 years old* 9th grade	Self-reported mathematics, mother-tongue (Finnish), humanities, and science grades (continuous)	Range = 16.5–19 years old	Youth offending as measured using official court data of criminal convictions (binary)	2–4 y*	7
(Smith et al., 2013)	USA	846	In phase 1 of data collection: 68% African American, 17% Hispanic and 15% White	72.9%	Range = 14–18 years old	Self-reported grades of participant’s overall letter grades at each interview, translated into a 5-point numeric scale (e.g. grade A = 5.) School GPA was taken from Rochester School data during the year 1989 (continuous)	Range = 21 to 23 years old	Delinquency/crime was measured from self-reported general crime (index of 25 offence types; continuous), self-reported violent crime (general crime index subscale containing 6 questions about violent interactions; continuous), official arrest data taken from Rochester Police Department records (binary), and self-reported perpetration of intimate partner violence data (continuous)	7–9 y*	8
(Zych et al., 2021)^†^	UK; Switzerland; USA	CSDD = 399* z-proso = 1274 PYS = 508	CSDD (London) = 97% White; z-proso (Zurich) = 81.8% White PYS (Pittsburgh) = 42.7% White. Other Ethnicity data were not provided for both samples	CSDD = 100%; z-proso = not reported; PYS = 100%	CSDD: range = 8–9 years old; z-proso: range = 7.5–9.5 years old; PYS: range = 10–11	CSDD: Administrative school records of arithmetic, English, and verbal reasoning; z-proso: teacher reported mathematics and language achievement (continuous); PYS: Mother, boy, and teacher-reported measure of achievement	CSDD = 10–16 years old; year 6–12*; z-proso = 12–17 years old; 6th–12th grade*; PYS = 10–16 years old; 4th–11th grade*	CSDD; z-proso: Official court records of convictions PYS: Petitions to the juvenile court (binary)	CSDD: 2–8 y* z-proso: 4.5–9.5 y* PYS: < 1–6 y*	6

*CSDD*, The Cambridge Study in Delinquent Development; *GCSE*, General Certificate of Secondary Education; *GPA*, Grade Point Average; *KYPS*, Korea Youth Panel Survey; *M*, mean; *PYS*, Pittsburgh Youth Study; *SD*, standard deviation; UK, United Kingdom; USA, United States of America; *z-proso*, Zurich Project on the Social Development from Childhood to Adulthood

Studies by Mercer et al. (2016), Farrington et al. (2016), and Zych et al. (2021) drew from the same sample of *n *= 411 boys in the UK. Defoe et al. (2013) and Karriker-Jaffe et al. (2021) also exclusively focused on male participants, whereas the remaining studies had more balanced gender representation intheir samples

All eligible studies drew participants from schools or school districts and assessed academic achievement during a period of compulsory education. Most studies relied on self-, parent-, or teacher-reports to gather exposure and outcome data, with only six studies using administrative school records to examine academic achievement and five studies using criminal records to examine youth offending. Followup periods ranged from less than 1 year to 11 years

Dodge et al.’s (2008) index of adolescent violent behaviour used six youth-report items from Huizinga and Elliot’s (1987) Self-Report of Delinquency, eight items from the Guns and Gangs instrument developed for this study (CPPRG, 1999), and two parent-report items from the Parent Report of Delinquency (CPPRG, 1999)

For Zych et al. (2021), only CSDD (London, UK) and z-proso (Zurich, Switzerland) samples were used in a meta-analysis

*Estimated from information provided by the article

†Study included in the meta-analysis

### Risk of Bias Assessment

The overall quality of eligible studies was moderate, with a mean NOS score of 7 out of 13. Of the 17 studies, only 6 met fewer than half of the assessed NOS quality criteria (Table 2, Table S3). Studies were particularly varied in how representative their samples were of the target population. Also, none of the eligible studies reported conducting a power analysis or otherwise justified their sample sizes, such that studies with smaller sample sizes may have been underpowered.

Many studies controlled for gender, age, or additional potential confounders, such as socio-economic status (SES), peer delinquency, parental education, and prior offending at baseline. However, when it came to reporting statistical results, most studies did not provide comprehensive information on relevant descriptive statistics and attrition rates. Also, most studies did not fully report effect estimates, *p*-values, and measures of precision.

## Meta-Analysis

A total of eight studies were included in the meta-analysis. Zych et al. (2021) used populations of participants from London and Zurich, which we included as separate estimates in a meta-analysis since they reported on cohorts from different studies. Farrington et al. (2016) drew from the same sample as Zych et al.’s (2021) London analysis but appeared to use data from slightly differing timepoints, so we included both studies in our meta-analysis; sensitivity analyses using cluster-robust variance estimation to account for these studies drawing from the same sample produced identical pooled point estimates to the analysis we report here.

**Fig. 2:**
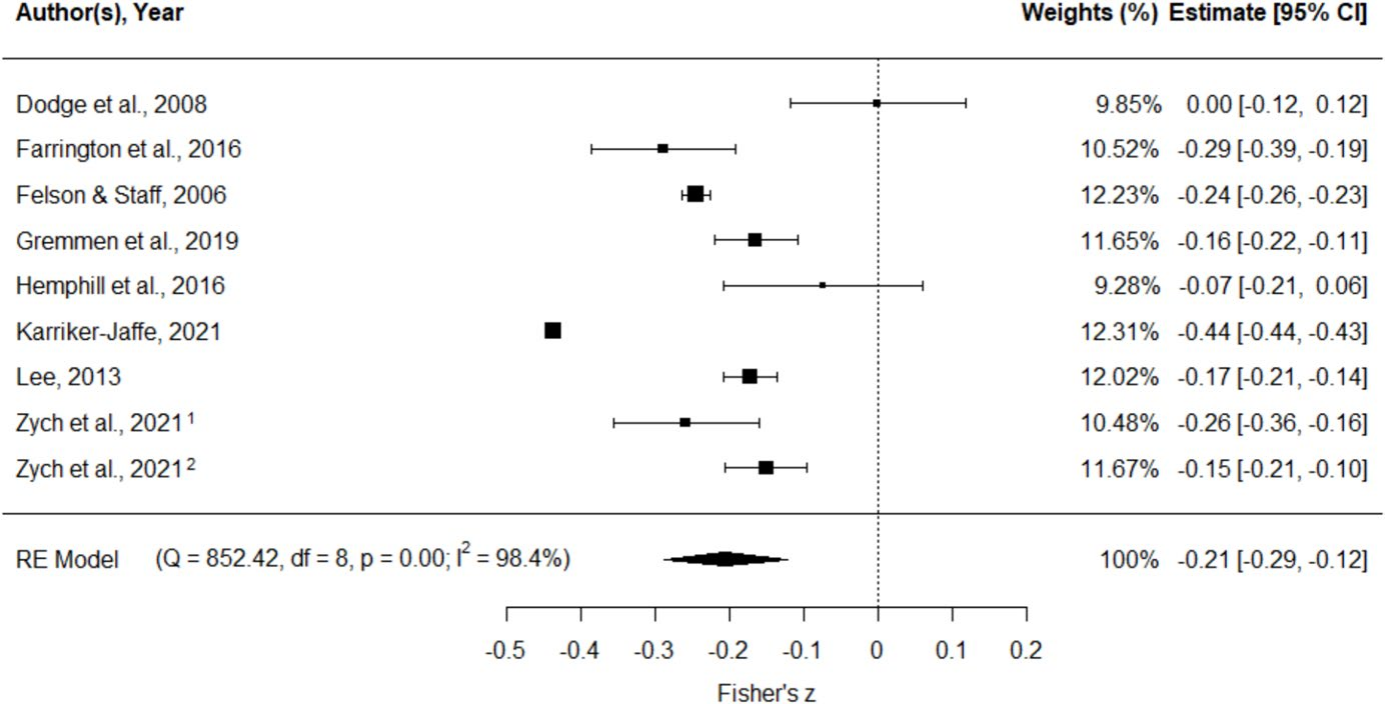
Meta-analysis of Fisher’s *z* between academic achievement and offending. Note: Study weights (%) are from random effects analysis. Zych et al. 2021^1^ is the London, UK (CSDD), sample. Zych et al. 2021^2^ is the Zurich, Switzerland (z-proso), sample

**Fig. 3:**
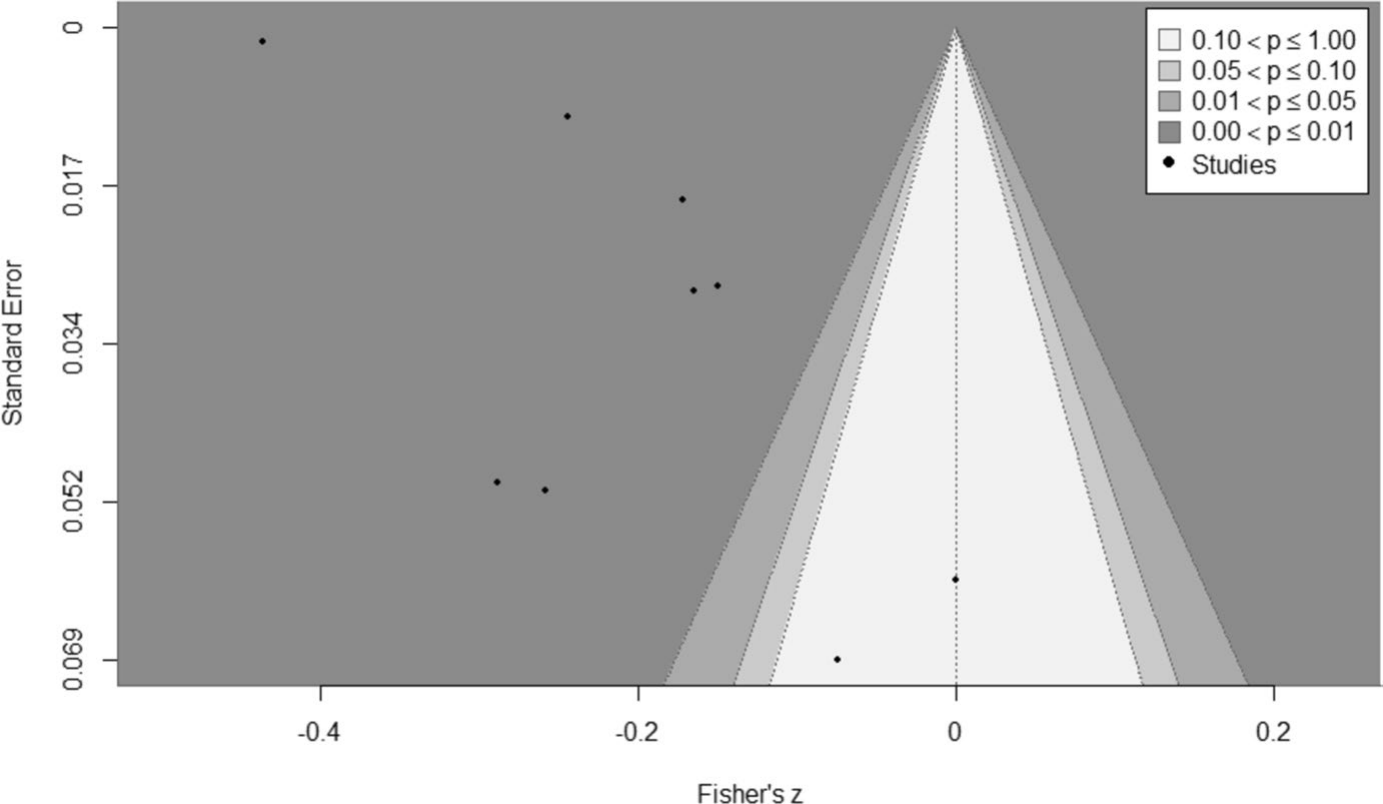
Funnel plot

We found a small but statistically significant association between lower academic achievement and increased youth offending; pooled Fisher’s *z* =  − 0.21, 95% CI [− 0.29, − 0.12], *p* < 0.001 (Fig. 2). This can be back-converted to a pooled correlation coefficient of *r* =  − 0.20. There was substantial and statistically significant heterogeneity between the studies (*I*^2^ = 98.4%; *Q* (8) = 852.4, *p* < 0.001). Egger’s test showed some evidence of publication bias (*p* = 0.030), and the funnel plot (Fig. 3) appears non-symmetric. Our subgroup analyses did not identify any strong or statistically significant effect modifiers (Table 3).

**Table 3:** Findings from subgroup analyses

Subgroup	Subgroup pooled Fisher’s z (95% CI), *p*-value	Difference between subgroups, Fisher’s *z* (95% CI), *p*-value
***Exposure measurement***		
* Administrative records (n* = *6)*	− 0.24 (− 0.35 to − 0.12), < *0.001*	0.08 (− 0.04 to 0.20), 0.199
* Self-/informant-report (n* = *3)*	− 0.16 (− 0.19 to − 0.13), < *0.001*	
***Outcome measurement***		
* Administrative records (n* = *4)*	− 0.29 (− 0.41 to − 0.16), < *0.001*	0.14 (− 0.01 to 0.29), 0.062
* Self-/informant-report (n* = *5)*	− 0.15 (− 0.23 to − 0.07), < *0.001*	
***Follow-up period***		
< *6 years (n* = *4)*	− 0.21 (− 0.26 to − 0.16), < *0.001*	− 0.02 (− 0.18 to 0.14), 0.802
≥ *6 years (n* = *5)*	− 0.19 (− 0.34 to − 0.04), *0.015*	

### Narrative Synthesis

Of the nine studies not included in the meta-analysis, eight studies found a statistically significant association between lower academic achievement and increased youth offending. However, Crosnoe (2002) only found this association to be significant in their non-college preparatory track, not in their college-preparatory track. One study reported an association in the opposite direction, with low academic achievement significantly predicting abstaining from delinquency compared to self-reported delinquency (Mercer et al., 2016). Across four studies which reported odds ratios (Farrington et al., 2016; Hemphill et al., 2016; Mercer et al., 2016; Zych et al., 2021), the reported adjusted odds ratios (which accounted for varying confounders) ranged from 1.31 (high drug use sample) to 3.24 (low family SES sample) (Hemphill et al., 2016).

Included studies reported a wide range of potential covariates and confounders (Table S2). Except for Hemphill et al. (2016), Dodge et al. (2008), Felson and Staff (2006), and Crosnoe (2002) (college-preparatory track), most of the studies still found a statistically significant association between lower academic achievement and increased youth offending after controlling for various covariates and confounders. Felson and Staff (2006) found this to be the case after controlling for various covariates in three of their models, but when attachment, commitment and effort were included in the model this became non-significant. Gremmen et al. (2019), in their network analysis, found that GPA only significantly negatively affected delinquency in 7th and 9th grades, not 8th grade. Farrington et al. (2016) found both that higher school attainment acted as a protective factor and lower school attainment as a risk factor. They also found that various factors moderated the effect of attainment, e.g. low ‘troublesomeness’ (extent of getting into trouble, as rated by peers and teachers) protected against the effect of low school attainment on offending. Furthermore, ten studies that appeared to adjust for prior offending also still found a statistically significant association between lower academic achievements and increased subsequent offending.

Regarding our secondary research question on offence types, one distinction made was between violent/aggressive offending (such as physical fighting and using a weapon during a fight) and non-violent/aggressive offending (such as shoplifting, painting graffiti and property damage) (Choi, 2007; Savage & Ellis, 2019). Both studies found a stronger association between lower academic achievement and violent/aggressive offending, compared to non-violent/non-aggressive offending; however, these associations were not statistically tested. In contrast, Sabates (2008) found a significant association between lower educational attainment and conviction rates for burglary, theft and vandalism, and drug offences, but a non-significant association for violent offending. Smith et al. (2013) made an additional distinction by categorising crime into official arrest records, self-reported perpetration of intimate partner violence, self-reported general crime (including minor offences such as public rowdiness, and serious offences, such as robbery and assault with a deadly weapon), and self-reported violent crime (encompassing specific items of the general crime index such as robbery, assault and gang fights). They found significant associations between lower school GPA and all categories of crime, with the relationship between school GPA and official arrest records being the strongest.

## Discussion

This meta-analysis provides comprehensive evidence for a weak but statistically significant association between lower academic achievement and increased risk for subsequent youth offending that appears to persist following adjustment for a number of confounders, such as prior offending. Included studies were very varied in their methodologies, although results did not appear to vary according to the source of exposure or outcome measurement, or the timing of these measurements, operationalised as the duration of follow-up. Our narrative synthesis indicated that the relationship may vary according to different offence types, but very few studies investigated this.

Our findings are similar to a previous meta-analysis by Savage et al. (2017) which focused on academic achievement and violent behaviour. By expanding our analysis to capture studies of criminal offending and delinquency in general, we capture a wider literature showing associations between lower academic achievement and youth offending. Our meta-analytic findings were also consistent with other longitudinal studies exploring the association between academic difficulties and future offending behaviour (Farrington, 2003; Martins et al., 2022), and in our narrative review, we found that these associations remained robust following adjustment for various potential confounders such as SES, parental and family characteristics, and substance and alcohol use. Our findings also support the ICAP theory’s suggestion that low academic achievement increases long-term antisocial potential (Farrington, 2018). Previous research shows how individual characteristics, such as self-control and sense of self, can moderate the impact of academic achievement on both short-term and long-term antisocial potential (AP). For example, Farrington et al. (2016) demonstrate how characteristics such as ‘low troublesomeness’ may act as a protective factor, reducing the risk of an individual offending regardless of low academic achievement. This is consistent with ICAP theory’s emphasis on the variable effect of risk and protective factors between individuals (Farrington, 2018).

Our narrative synthesis showed mixed findings on the observed association for different offence types. Some studies tentatively suggested a stronger association between achievement and violent/aggressive offending compared to non-violent/aggressive offending, consistent with Savage et al. (2017). However, Sabates (2008) found a non-significant association between educational achievement and violent offending, compared to significant associations for all other investigated offence types. As such, findings on the differential association between academic achievement and violent and non-violent offending remain equivocal and warrant further investigation into offence-specific pathways. Examining factors including impulsivity, social pressures, and societal norms, is therefore crucial, as these variables have the potential to help identify the distinct mechanisms that underpin the relationship between academic achievement and different offending behaviour (Hoffmann, 2018; Zhu et al., 2024). By addressing wider social, educational, and personality factors, this offers an opportunity to broaden the application of the ICAP theory to account for variability across different offence types.

A strength of this study was the use of a comprehensive search strategy applied to relevant electronic databases. We aligned keywords and subject headings closely to our research question to capture a large number of relevant studies that had not been captured by a previous meta-analysis in this area (Savage et al., 2017). We also took a robust approach to screening, data extraction, and risk of bias assessment with two independent reviewers. This supported our aim to produce as robust an estimate of the association between academic achievement and offending as possible.

This study also has some limitations. Many included studies relied on self-reported measures, which may carry biases for measuring delinquency or attainment. Studies using self-reported offending measures may report higher offending rates than official records (Piquero et al., 2014; Pollock et al., 2015); however, there is generally moderate agreement between official and self-reported measures of offending (Piquero et al., 2014). The validity of self-reported attainment is also questionable and has been shown to vary according to students’ ability; for example, children with lower achievement may be more likely to self-report grades inaccurately (Kuncel et al., 2005; Teye & Peaslee, 2015). Additionally, although our focus on longitudinal studies with prospective data collection allowed for better inference of a direction of association, some of the studies took approaches to longitudinal modelling which could not be incorporated into our meta-analysis. Our research also showed evidence of publication bias, and the exclusion of studies not published in the English language may have resulted in the omission of important research findings. This limitation also restricts the generalisability of our findings to countries not captured by this research. Our research predominantly captured eligible studies that were carried out in high-income North American or European countries. Furthermore, studies were of moderate quality overall and exhibited significant heterogeneity; our sub-group analyses did not identify any statistically significant study-level effect modifiers, such that understanding modifiers underlying this heterogeneity would be an important area for future research.

In addition to offending outcomes, low academic achievement can have an extensive impact throughout an individual’s life, contributing towards various negative outcomes such as unemployment, homelessness, poor health, and suicide (Almquist, 2013; Brakenhoff et al., 2015; Caspi et al., 1998; Sörberg Wallin et al., 2018). Our research, alongside insights provided by Savage et al. (2017), demonstrates that lower academic achievement is associated with subsequent youth offending. This may have significant implications for youth and adult custody services and the education sector, particularly as youth justice practitioners navigate the recent shift away from risk-centric towards child-first approaches (Day, 2023).

Tentatively, our findings suggest that child-first prevention approaches incorporating strategies to improve academic achievement may be beneficial, although further work establishing a causal pathway would be required. In keeping with the ICAP theory, it remains plausible that interventions to improve academic achievement could disrupt the pathway from low attainment to antisocial behaviour, particularly if tackled alongside other risk factors. Additionally, low or declining levels of academic attainment may be a readily available marker for schools to identify pupils who are potentially ‘at-risk’ (Wickersham, 2024) and may benefit from educational interventions as well as behavioural and emotional skills interventions (Farrington et al., 2017).

It should be acknowledged that achievement is just one aspect of the school journey and that other educational factors may also play an important role in this pathway, such as school absence and exclusion. By enhancing our understanding of the pathways from academic achievement to offending, and taking into account other educational factors, we can inform research-based initiatives aimed at promoting educational success, which might in turn reduce subsequent rates of offending and re-offending. Because education is compulsory for all young people in many countries, schools could present an ideal setting to identify pupils at increased risk of offending and offer appropriate support before problems escalate (Melendez-Torres et al., 2023)—this could be the subject of further experimental trials.

Future research must examine how sociodemographic characteristics, other education factors, and social care impact the association between academic achievement and youth offending, especially for youth ‘at-risk’ of later offending. Identifying critical areas of intervention, such as early educational challenges, school absences, or exclusion, could enable targeted efforts to enhance student engagement and offer specialised social support (Melendez-Torres et al., 2023). Schools and other education services could implement early interventions which address the academic and psychosocial needs of at-risk youth by employing ICAP-informed practices. This has the potential to inform youth justice practices and educational policies that contribute to the broader application of comprehensive, child-focused approaches, with the aim of reducing youth offending rates.

Although some included studies measured academic achievement and offending at multiple timepoints, future longitudinal studies could helpfully establish periods during an individual’s academic career when changes in academic achievement most strongly associated with offending outcomes, potentially highlighting key timepoints for intervention. Methodological considerations would play an important role in designing such future studies; for example, while we found no modifying effect of where exposure and outcome measures were sourced from, it is notable that the two studies in our meta-analysis which found the weakest association between academic achievement and offending were also the only two studies which measured achievement on binary scales, rather than continuous scales or categorical scales with more categories (Dodge et al., 2008; Hemphill et al., 2016). Therefore, measuring achievement using more granular scales potentially affords greater sensitivity for detecting such associations. Future studies could also conduct more comprehensive investigations examining the role of potentially modifying factors, including various sociodemographic, educational and social care characteristics, and the impact on different offence types. This may yield more specific recommendations for front-line practice and for policy.

In conclusion, we conducted a comprehensive meta-analysis and narrative synthesis on the association between lower academic achievement and later youth offending. We found evidence for a small but statistically significant association. Further investigations into this complex association are needed to establish early prevention and intervention initiatives that address the specific challenges and needs of individuals most at risk of later offending.

## Supplementary Information

Below is the link to the electronic supplementary material.

ESM 1 (DOCX 56.7 KB)

## Data Availability

Data used in the meta-analysis and accompanying statistical code will be made available on ML’s GitHub on publication.
