# Supplementary material for: The Association Between Academic Achievement and Subsequent Youth Offending: A Systematic Review and Meta-Analysis
Source: J Dev Life Course Criminol. 2025 Mar 20;10(4):477–500. doi: 10.1007/s40865-025-00266-9 (PMC12102114; doi:10.1007/s40865-025-00266-9)
Supplement: Supplementary file 1 — ESM 1 (DOCX 56.7 KB) [file 40865_2025_266_MOESM1_ESM.docx]

| **Section and Topic** | **Item #** | **Checklist item** | **Location where item is reported** |
| --- | --- | --- | --- |
| **TITLE** | | |  |
| Title | 1 | Identify the report as a systematic review. | 1 |
| **ABSTRACT** | | |  |
| Abstract | 2 | See the PRISMA 2020 for Abstracts checklist. | 1 |
| **INTRODUCTION** | | |  |
| Rationale | 3 | Describe the rationale for the review in the context of existing knowledge. | 2-3 |
| Objectives | 4 | Provide an explicit statement of the objective(s) or question(s) the review addresses. | 3 |
| **METHODS** | | |  |
| Eligibility criteria | 5 | Specify the inclusion and exclusion criteria for the review and how studies were grouped for the syntheses. | 4-5 |
| Information sources | 6 | Specify all databases, registers, websites, organisations, reference lists and other sources searched or consulted to identify studies. Specify the date when each source was last searched or consulted. | 5 |
| Search strategy | 7 | Present the full search strategies for all databases, registers and websites, including any filters and limits used. | 5 |
| Selection process | 8 | Specify the methods used to decide whether a study met the inclusion criteria of the review, including how many reviewers screened each record and each report retrieved, whether they worked independently, and if applicable, details of automation tools used in the process. | 5-6 |
| Data collection process | 9 | Specify the methods used to collect data from reports, including how many reviewers collected data from each report, whether they worked independently, any processes for obtaining or confirming data from study investigators, and if applicable, details of automation tools used in the process. | 6 |
| Data items | 10a | List and define all outcomes for which data were sought. Specify whether all results that were compatible with each outcome domain in each study were sought (e.g. for all measures, time points, analyses), and if not, the methods used to decide which results to collect. | 5-6 |
|  | 10b | List and define all other variables for which data were sought (e.g. participant and intervention characteristics, funding sources). Describe any assumptions made about any missing or unclear information. | 6 |
| Study risk of bias assessment | 11 | Specify the methods used to assess risk of bias in the included studies, including details of the tool(s) used, how many reviewers assessed each study and whether they worked independently, and if applicable, details of automation tools used in the process. | 6 |
| Effect measures | 12 | Specify for each outcome the effect measure(s) (e.g. risk ratio, mean difference) used in the synthesis or presentation of results. | 6-7 |
| Synthesis methods | 13a | Describe the processes used to decide which studies were eligible for each synthesis (e.g. tabulating the study intervention characteristics and comparing against the planned groups for each synthesis (item #5)). | 6-7 |
|  | 13b | Describe any methods required to prepare the data for presentation or synthesis, such as handling of missing summary statistics, or data conversions. | 6-7 |
|  | 13c | Describe any methods used to tabulate or visually display results of individual studies and syntheses. | 6-7 |
|  | 13d | Describe any methods used to synthesize results and provide a rationale for the choice(s). If meta-analysis was performed, describe the model(s), method(s) to identify the presence and extent of statistical heterogeneity, and software package(s) used. | 6-7 |
|  | 13e | Describe any methods used to explore possible causes of heterogeneity among study results (e.g. subgroup analysis, meta-regression). | 7 |
|  | 13f | Describe any sensitivity analyses conducted to assess robustness of the synthesized results. | N / A |
| Reporting bias assessment | 14 | Describe any methods used to assess risk of bias due to missing results in a synthesis (arising from reporting biases). | N / A |
| Certainty assessment | 15 | Describe any methods used to assess certainty (or confidence) in the body of evidence for an outcome. | N / A |
| **RESULTS** | | |  |
| Study selection | 16a | Describe the results of the search and selection process, from the number of records identified in the search to the number of studies included in the review, ideally using a flow diagram. | 9 |
|  | 16b | Cite studies that might appear to meet the inclusion criteria, but which were excluded, and explain why they were excluded. | N/A |
| Study characteristics | 17 | Cite each included study and present its characteristics. | 10-17 |
| Risk of bias in studies | 18 | Present assessments of risk of bias for each included study. | 18 |
| Results of individual studies | 19 | For all outcomes, present, for each study: (a) summary statistics for each group (where appropriate) and (b) an effect estimate and its precision (e.g. confidence/credible interval), ideally using structured tables or plots. | 19 |
| Results of syntheses | 20a | For each synthesis, briefly summarise the characteristics and risk of bias among contributing studies. | 20-21 |
|  | 20b | Present results of all statistical syntheses conducted. If meta-analysis was done, present for each the summary estimate and its precision (e.g. confidence/credible interval) and measures of statistical heterogeneity. If comparing groups, describe the direction of the effect. | 19 |
|  | 20c | Present results of all investigations of possible causes of heterogeneity among study results. | 19 |
|  | 20d | Present results of all sensitivity analyses conducted to assess the robustness of the synthesized results. | 18 |
| Reporting biases | 21 | Present assessments of risk of bias due to missing results (arising from reporting biases) for each synthesis assessed. | N/A |
| Certainty of evidence | 22 | Present assessments of certainty (or confidence) in the body of evidence for each outcome assessed. | N/A |
| **DISCUSSION** | | |  |
| Discussion | 23a | Provide a general interpretation of the results in the context of other evidence. | 22-24 |
|  | 23b | Discuss any limitations of the evidence included in the review. | 23 |
|  | 23c | Discuss any limitations of the review processes used. | 23 |
|  | 23d | Discuss implications of the results for practice, policy, and future research. | 22-24 |

From: Page MJ, McKenzie JE, Bossuyt PM, Boutron I, Hoffmann TC, Mulrow CD, et al. The PRISMA 2020 statement: an updated guideline for reporting systematic reviews. BMJ 2021;372:n71. doi: 10.1136/bmj.n71

For more information, visit: <http://www.prisma-statement.org/>

The following tables demonstrate the searches run for the PsycINFO, ERIC, BEI, and Web of Science databases, which were developed from a broader search term strategy and adapted according to the thesaurus of each database.

**PsycINFO via Ovid, searched 26/10/2021 (APA PsycInfo 18/06 to Week 3 in Oct 2021)**

|  | child*.tw OR adolescen*.tw OR teenag*.tw OR youth*.tw OR young.tw |
| --- | --- |
| AND | exp academic achievement/ OR exp academic failure/ OR exp educational attainment level/ OR exp grade level/ OR ((academic or educational or school or classroom) adj (achievement or performance or attainment or success or failure)).tw |
| AND | exp crime/ OR exp criminal behavior/ OR exp criminal conviction/ OR exp criminal offenders/ OR exp criminal record/ OR exp juvenile delinquency/ OR crim*.tw OR offen*.tw OR felon*.tw OR misdemeanour.tw OR illegal.tw OR unlawful.tw OR convict*.tw OR delinquen*.tw |

**Results: 2,131**

**ERIC via EBSCO, searched 26/10/2021**

|  | [Explode "Adolescents", "Children", "Early Adolescents", "Late Adolescents", "Youth", “Young Adults”, which results in below DE string]  DE "Adolescents" OR DE "Children" OR DE "African American Children" OR DE "Grandchildren" OR DE "Hospitalized Children" OR DE "Latchkey Children" OR DE "Migrant Children" OR DE "Minority Group Children" OR DE "Preadolescents" OR DE "Young Children" OR DE "Early Adolescents" OR DE "Late Adolescents" OR DE "Youth" OR DE "Disadvantaged Youth" OR DE "Out of School Youth" OR DE "Rural Youth" OR DE "Urban Youth" OR DE "Young Adults"  OR TI “child*” OR TI “adolescen*” OR TI “teenag*” OR TI “youth*” OR TI “young”  OR AB “child*” OR AB “adolescen*” OR AB “teenag*” OR AB “youth*” OR AB “young” |
| --- | --- |
| AND | [Explode "Achievement", "Failure", "Grades (Scholastic)", "Student Evaluation", which results in below DE string]  DE "Achievement" OR DE "Academic Achievement" OR DE "African American Achievement" OR DE "Graduation" OR DE "High Achievement" OR DE "Knowledge Level" OR DE "Low Achievement" OR DE "Mathematics Achievement" OR DE "Overachievement" OR DE "Reading Achievement" OR DE "Scholarship" OR DE "Science Achievement" OR DE "Underachievement" OR DE "Writing Achievement" OR DE "Failure" OR DE "Academic Failure" OR DE "Grades (Scholastic)" OR DE "Grade Inflation" OR DE "Grade Point Average" OR DE "Student Evaluation" OR DE "Curriculum Based Assessment" OR DE "Nongraded Student Evaluation" OR DE "Progress Monitoring"  OR TI “academic achievement” OR TI “academic performance” OR TI “academic attainment” OR TI “academic success” OR TI “academic failure” OR TI “educational achievement” OR TI “educational performance” OR TI “educational attainment” OR TI “educational success” OR TI “educational failure” OR TI “school achievement” OR TI “school performance” OR TI “school attainment” OR TI “school success” OR TI “school failure” OR TI “classroom achievement” OR TI “classroom performance” OR TI “classroom attainment” OR TI “classroom success” OR TI “classroom failure”  OR AB “academic achievement” OR AB “academic performance” OR AB “academic attainment” OR AB “academic success” OR AB “academic failure” OR AB “educational achievement” OR AB “educational performance” OR AB “educational attainment” OR AB “educational success” OR AB “educational failure” OR AB “school achievement” OR AB “school performance” OR AB “school attainment” OR AB “school success” OR AB “school failure” OR AB “classroom achievement” OR AB “classroom performance” OR AB “classroom attainment” OR AB “classroom success” OR AB “classroom failure” |
| AND | [Explode “Crime”, which results in below DE string]  DE “Crime” OR DE “Delinquency”  OR TI “crim*” OR TI “offen*” OR TI “felon*” OR TI “misdemeanour” OR TI “illegal” OR TI “unlawful” OR TI “convict*” OR TI “delinquen*”  OR AB “crim*” OR AB “offen*” OR AB “felon*” OR AB “misdemeanour” OR AB “illegal” OR AB “unlawful” OR AB “convict*” OR AB “delinquen*” |

**Results: 1,290**

**BEI via EBSCO, searched 26/10/2021**

|  | exp children OR exp adolescence OR exp youth OR exp young adults which comes out as:  DE "CHILDREN" OR DE "ABUSED children" OR DE "ADOPTED children" OR DE "BIRTH order" OR DE "BLACK children" OR DE "BOYS" OR DE "CHILD development" OR DE "CHILDREN as teachers" OR DE "CHILDREN of attention-deficit-disordered parents" OR DE "CHILDREN of divorced parents" OR DE "CHILDREN of foreign workers" OR DE "CHILDREN of immigrants" OR DE "CHILDREN of migrant laborers" OR DE "CHILDREN of minorities" OR DE "CHILDREN of older parents" OR DE "CHILDREN of parents with disabilities" OR DE "CHILDREN of school principals" OR DE "CHILDREN of single parents" OR DE "CHILDREN of teenage mothers" OR DE "CHILDREN of the rich" OR DE "CHILDREN of unmarried parents" OR DE "CHILDREN of working parents" OR DE "CHILDREN'S television programs" OR DE "CITY children" OR DE "EXCEPTIONAL children" OR DE "FOSTER children" OR DE "GIRLS" OR DE "GRANDCHILDREN" OR DE "HANDICRAFT for children" OR DE "HOMELESS children" OR DE "MENTALLY ill children" OR DE "PLAYMATES" OR DE "POOR children" OR DE "PRESCHOOL children" OR DE "PROBLEM children" OR DE "REFUGEE children" OR DE "RELIGIOUS education of children" OR DE "SCHOOL children" OR DE "ADOLESCENCE" OR DE "YOUTH" OR DE "AT-risk youth" OR DE "BISEXUAL youth" OR DE "BLACK youth" OR DE "JUVENILE delinquents" OR DE "LGBTQ+ youth" OR DE "MENTALLY ill youth" OR DE "MINORITY youth" OR DE "POOR youth" OR DE "PROBLEM youth" OR DE "RELIGIOUS education of young people" OR DE "SCHOOL dropouts" OR DE "TEENAGERS" OR DE "URBAN youth" OR DE "YOUNG adults" OR DE "YOUTH with disabilities" OR DE "YOUNG adults" OR DE "YOUNG men" OR DE "YOUNG women"  OR TI “child*” OR TI “adolescen*” OR TI “teenag*” OR TI “youth*” OR TI “young”  OR AB “child*” OR AB “adolescen*” OR AB “teenag*” OR AB “youth*” OR AB “young” |
| --- | --- |
| AND | exp academic achievement OR exp school failure which comes out as:  DE "ACADEMIC achievement" OR DE "ACADEMIC motivation" OR DE "ACADEMIC overachievement" OR DE "ACADEMIC underachievement" OR DE "ACHIEVEMENT gap" OR DE "ACHIEVEMENT tests" OR DE "BIRTH date effect (Academic achievement)" OR DE "COMPOSITION (Language arts) achievement" OR DE "COMPREHENSIVE examinations" OR DE "EDUCATIONAL attainment" OR DE "GRADUATION (Education)" OR DE "PERSONALITY & academic achievement" OR DE "PREDICTION of scholastic success" OR DE "SCHOOL failure"  OR TI “academic achievement” OR TI “academic performance” OR TI “academic attainment” OR TI “academic success” OR TI “academic failure” OR TI “educational achievement” OR TI “educational performance” OR TI “educational attainment” OR TI “educational success” OR TI “educational failure” OR TI “school achievement” OR TI “school performance” OR TI “school attainment” OR TI “school success” OR TI “school failure” OR TI “classroom achievement” OR TI “classroom performance” OR TI “classroom attainment” OR TI “classroom success” OR TI “classroom failure”  OR AB “academic achievement” OR AB “academic performance” OR AB “academic attainment” OR AB “academic success” OR AB “academic failure” OR AB “educational achievement” OR AB “educational performance” OR AB “educational attainment” OR AB “educational success” OR AB “educational failure” OR AB “school achievement” OR AB “school performance” OR AB “school attainment” OR AB “school success” OR AB “school failure” OR AB “classroom achievement” OR AB “classroom performance” OR AB “classroom attainment” OR AB “classroom success” OR AB “classroom failure” |
| AND | DE "CRIMINOLOGY" OR DE “DELINQUENT behavior”  OR TI “crim*” OR TI “offen*” OR TI “felon*” OR TI “misdemeanour” OR TI “illegal” OR TI “unlawful” OR TI “convict*” OR TI “delinquen*”  OR AB “crim*” OR AB “offen*” OR AB “felon*” OR AB “misdemeanour” OR AB “illegal” OR AB “unlawful” OR AB “convict*” OR AB “delinquen*” |

**Results: 29**

**Web of Science Core Collection, searched 26/10/2021**

|  | (TI=(“child*” OR “adolescen*” OR “teenag*” OR “youth*” OR “young”)  OR AB=(“child*” OR “adolescen*” OR “teenag*” OR “youth*” OR “young”)  OR TS=(“child*” OR “adolescen*” OR “teenag*” OR “youth*” OR “young”)) |
| --- | --- |
| AND | (TI=((academic or educational or school or classroom) NEAR/0 (achievement or performance or attainment or success or failure))  OR AB=((academic or educational or school or classroom) NEAR/0 (achievement or performance or attainment or success or failure))  OR TS=((academic or educational or school or classroom) NEAR/0 (achievement or performance or attainment or success or failure))) |
| AND | (TI=(“crim*” OR “offen*” OR “felon*” OR “misdemeanour” OR “illegal” OR “unlawful” OR “convict*” OR “delinquen*”)  OR AB=(“crim*” OR “offen*” OR “felon*” OR “misdemeanour” OR “illegal” OR “unlawful” OR “convict*” OR “delinquen*”)  OR TS=(“crim*” OR “offen*” OR “felon*” OR “misdemeanour” OR “illegal” OR “unlawful” OR “convict*” OR “delinquen*”)) |

**Results: 1,280**

**Note.**

- “.tw” denotes searching the table of contents, title, abstract, and key concepts in PsycINFO. “TI” and “AB” denotes keyword searching in the title and abstract for BEI and ERIC.
- “exp XYZ/” denotes exploding a subject heading in PsycINFO. In BEI and ERIC, this is achieved with “DE”.
- * denotes truncation.

Note: A study can be awarded a maximum of one star for each numbered item within the Selection and Outcome categories. A maximum of two stars can be given for Comparability.

**Selection (maximum 5)**

1. Representativeness of the exposed cohort
2. Truly representative of the average in the target population (e.g., random sample or whole population.) *
3. Somewhat representative of the average in the target population (e.g., purposive sampling of representative schools or evidence that the sample is representative of the source population.) *
4. Selected group may not be representative of the average in the target population.
5. Derivation of the cohort not adequately described.
6. Selection of the non-exposed cohort
7. Drawn from the same community as the exposed cohort. *
8. Drawn from a different source.
9. No description of the derivation of the non-exposed cohort.
10. Ascertainment of exposure (educational attainment)
11. Administrative records or a named measurement instrument. *
12. Reported by teacher, parent or other informant.
13. No description.
14. Adjustment made for baseline or prior offending in multivariable analysis
15. Yes *
16. No
17. Sample size
18. Justified and satisfactory. *
19. Not justified.

**Comparability (maximum 2)**

1. Comparability of cohorts on the basis of the design or analysis
2. Study controls for age and gender (either in selection of the cohort, or in adjusted/stratified analyses.) *
3. Study controls for any additional factor. *
4. Study does not control for any factors.

**Outcome (maximum 4)**

1. Assessment of outcome (criminal offending)
2. Administrative records or a named measurement instrument. *
3. Reported by teacher, parent or other informant.
4. No description.
5. Was follow-up long enough for outcomes to occur
6. Yes (≥1 year) *
7. No
8. Adequacy of follow-up of cohorts
9. Complete follow-up - all subjects accounted for. *
10. Subjects lost to follow-up unlikely to introduce bias - small number lost (<20%) or Attrition described and accounted for in analysis. *
11. Follow-up rate inadequate (>20%) and no description of those lost or accounting for those lost in analysis.
12. No statement.
13. Statistical test
14. The statistical test(s) used to analyse the data is clearly described, appropriate and complete, including a named effect estimate, a *p-value*, and a measure of precision if appropriate (i.e., standard errors for beta coefficients, confidence intervals for odds ratios.) *
15. The statistical test is not appropriate, not described or incomplete.

| **Lead Author, Year** | **Additional inclusion/exclusion criteria reported at the participant level** | **Offending/delinquency types captured** | **Covariates adjusted for (including unspecified covariates)** |
| --- | --- | --- | --- |
| Choi, 2007 | Participation in both waves 1 and 2.  Analysis omitted Native Americans (n = 115) and those of ‘other’ racial groups (n = 192). | Aggressive offences: threatening someone with a weapon, taking part in a group fight, pulling a knife or gun on someone, and shooting or stabbing someone. Non-aggressive offences: painting graffiti, damaging property, stealing something worth less than $50, shoplifting, stealing a car, stealing something worth more than $50, burglarising a building, and running away from home. | Age, gender, racial and ethnic groups, parental education and early problem behaviour measured in wave 1. |
| Crosnoe, 2002 | Participants had to have participated at 3 of the first 4 time points of data collection, had to have friendship network information from year 1 available and had to have reported track location information. | Taken something of value from another person, run away from home, gotten into trouble with the police, started a fight at school, carried a weapon to school, or purposely damaged school property. | Gender, family structure (intact vs. non-intact), age (controlled for through sample selection) and 2 ethnic dummy variables (African American or Hispanic American, Asian American), year 1 delinquency. Also, a dummy variable for being from Wisconsin to control for regional differences. |
| Defoe, 2013 | Participants had to be all males and had to score in the upper 30% on behaviour on the screening assessment. | Carrying a weapon, vandalism, firesetting, avoiding paying (e.g. a fare), breaking and entering, stealing an item worth less than $5, stealing an item worth $5-$50, stealing an item worth $50-$100, stealing an item worth more than $100, shoplifting, pickpocketing, stealing from a car, handling stolen goods, joyriding, vehicle theft, check fraud, credit card fraud, cheating someone out of money, attacking to hurt, robbery, gang fighting, hurting someone to get sex, forcing someone to have sex, selling marijuana, and selling heroin, cocaine, or LSD. | Gender (controlled for through sample selection), delinquency at t-1 |
| Dodge, 2008 | Participants had to have attended the control schools (Durham, North Carolina; Nashville, Tennessee; Seattle, Washington State; and central Pennsylvania) of a longitudinal, multisite investigation of  the development and prevention of conduct problems in children, the fast-track project. | Self-report: attacked anyone with intent to hurt/ kill, hit anyone with the idea of hurting them, used a weapon to get money from people, thrown objects at people, involved in any gang fights, had sex with someone against their will, pulled a small pocket knife on another person, hurt someone with a small pocket knife, pulled a switchblade, razor or other big knife on another person, hurt someone with a switchblade, razor or other big knife, pulled a gun (that your parent own for their protection or for hunting) on another person, hurt someone with a gun (that your parent own for their protection or for hunting), pulled a gun (that is not for hunting) on another person, hurt someone with a gun (that is not for hunting).  Parent report: has your child attacked someone with a weapon with the idea of seriously hurting them, has your child carried a weapon. | Early adverse social context, early harsh and inconsistent parenting, school social and cognitive readiness, early child externalizing behaviour, school social failure, adolescent parental monitoring, adolescent deviant peer association |
| Farrington, 2016 | Participants had to be male and belong to the second forms of 6 state primary schools in a working-class area of London. | Not Reported. | Gender (controlled for through sample selection), age, nonverbal and verbal intelligence, daring, hyperactivity, impulsiveness, extraversion, neuroticism, nervousness, popularity, number of friends, dishonesty, troublesomeness, family income, social class, housing family size, school delinquency rate, convicted parent, age of mother, nervousness of mother, job of mother, interest in education, delinquent sibling, child-rearing, supervision and separations. |
| Felson & Staff, 2006 | Participants included those who responded to the initial survey (1988; 8th grade) and the first 2 waves (1990 10th grade; 1992 12th grade) and who had official transcripts of their grades. | Underage drinking, drug misuse, physical fighting, and arrest. | Age (controlled for through sample selection) gender, race and ethnicity (black, Hispanic, Asian, American Indian, or white), and socioeconomic status (based on measures of father’s education level, mother’s education level, father’s occupation, mother’s occupation, and family income obtained from the 1988 parents’ survey), social bonds, self-control, earlier delinquency |
| Gremmen, 2019 | Participants had to be 1^st^ or 2^nd^ year high school students in 2011-2012 or 1^st^ year students in 2012-2013 cohorts (of the longitudinal project SNARE, Social Network Analysis of Risk behavior in Early adolescence) | Delinquency as measured according to these antisocial behaviours: Stealing, vandalism, burglary, violence, weapon carrying, threatening to use a weapon, truancy, contact with the police, and fare evasion in public transport. | Age, gender, track, alcohol use and parameter estimates as defined in the RSiena Model. Including both network dynamics (structural network and behaviour-dependent selection dynamics) and behaviour dynamics (behaviour tendencies and influence effects). |
| Hemphill, 2016 | Participants were part of a larger International Development Study in Victoria, Australia and Washington State, US. But only Victoria, Australia were used. | Beating someone up so badly that they needed medical attention, attacking someone with the intention of hurting them, threatening someone with a weapon | Age, gender, and the clustering of students in schools. |
| Karriker-Jaffe, 2021 | Participants had to be born between 1972-1982, with at least one biological parent registered and an assessment of school achievement and a valid assessment of resilience in the conscript register | Aggravated assault; illegal threats, intimidation, and coercion; threats or violence against a police officer; aggravated robbery; murder, manslaughter, or filicide; kidnapping; arson; sexual crimes (excluding prostitution and the buying of sexual services, but including child pornography); fraud, forgery, and embezzlement; and property crimes (theft, vandalism, vandalism causing danger to the public, and trespassing) | Gender and age (both controlled for through sample selection), Prior criminal behaviour |
| Lee, 2013 | Participants took part in the National Survey launched in 2003 and excluded students in Cheju Island and not in their 2^nd^ year of middle school (8^th^ grade) | Delinquent behaviours from the Korea Youth Panel Survey, including smoking, drinking, truancy, running away from home, sexual relationships, beating others severely, group fighting, blackmailing, stealing, adolescent prostitution, bullying or harassing, intimidating others, ostracizing other friends, and sexual violence or sexual harassment. | Age (controlled for through sample selection), delinquency and academic achievement at t-1, and measurement error in the autoregressive model analysis. |
| Lesner, 2022 | Born between 1996-2002, living in Denmark 16, attended public schools, started in grade 9 without delay, in school for the first eight months of the school year, only students employed at some point in the first eight months of grade 9 | Includes violence and sex offences, offences against property, drug related crime | Work experience (hours) first 8 months of grade 9 (standardised), work experience prior to grade 9, school absenteeism (grade 8), criminal charge (grade 8), Grade Point Average (GPA) from national tests (at grade 6/8), gender, age in months, industry, graduation year, parental characteristics |
| Mercer, 2016 | Participants had to be male and on the registers of 6 state primary schools within a one-mile radius of the research office that had been established. | Self-report: burglary, shoplifting, theft from motor vehicles, theft of motor vehicles, theft from machines, shoplifting and vandalism (+ started fights and ever used drugs for the age 18 interview)  Convicted: burglary, shoplifting, theft from motor vehicles, theft of motor vehicles, theft from machines, violent offences, vandalism and drug offences, as well as 10 other offences: fraud, theft from work, other theft, robbery, suspected persons, weapons offences, receiving offences, serious motor vehicle offences, threatening and sexual offences recorded from age 10–18. | Gender (controlled for through sample selection), disobedience, combined peer ratings of popularity, non-verbal IQ, combined ratings of daring, peer ratings of honesty, psychomotor impulsivity, concern with trying to be a credit to his parents, overall nervousness, New Junior Maudsley Inventory (NJMI) extraversion, NJMI neuroticism, NJMI social conformity and lacking concentration. |
| Sabates, 2008 | The selection of cohorts is based on the availability of aggregate data on conviction rates, educational attainment, poverty, time away from school, and school resources. Due to data coming from different sources, empirical analysis was restricted to three cohorts of individuals born 1981 - 1983. | Violent crime (defined according to Offenders Index data as murder, cruelty to children, child abduction, kidnapping, rioting, endangering wildlife or sea life, endangering railway passengers, and procuring illegal abortion), burglary, theft, criminal damage (including arson), and drug-related (misuse - both supply and use) offences.  *Note:* criminal damage and drug-related offences were merged into one category as a high proportion of young people who commit vandalism also commit drug offences by drug misuse or drug supply. | Age, percentage (%) of unauthorised half-day missed by local education authority (LEA), average pupil: teacher ratio in secondary schools, free school meals (FSM) eligibility, average conviction rate, average trend, between LEA variability in average conviction rate and trend. |
| Savage & Ellis, 2019 | Participants had to be 13 – 18 years old in wave 2, to ensure they were school-aged in the previous wave. | Violent offending - Got into a serious physical fight, took part in a fight "where a group of your friends was against another group", used or threatened to use a weapon to get something from someone, hurt someone badly enough to need medical care, pulled knife or gun on someone, used a weapon in a fight, Shot or stabbed someone. Nonviolent offending - damaged property, painted graffiti, stolen something worth more than US$50, sold marijuana or other drugs, stolen something worth less than US$50, taken something from a store without paying for it, went into a house to steal something | Age, gender, disadvantaged minority status, intact family or not (defined as living with both biological mother and father), neighbourhood disorder, parent education, peer delinquency, intelligence (using the Add Health Picture Vocabulary Test), attention deficits (ADHD), alcohol use and drug use. |
| Savolainen, 2012 | Individuals born between July 1, 1985 and June 30, 1986 in the two northern-most provinces of Finland (Hurtig et al., 2007). The study is limited to members of the birth cohort who participated in the adolescent self-report survey. | Not Reported. | Gender, age (controlled for through sample selection), low parent education, childhood antisociality (conduct problem score and hyperactivity score), learning difficulties (maths, reading and writing), delinquent involvement (delinquent peers, substance use, drug offers, drug use, intoxication frequency), family bonding (family meals, parental interest, family time) and school attachment. |
| Smith, 2013 | Participants had to attend Rochester, New York public schools in 1988. | General crime including 25 offences such as minor offences – e.g. public rowdiness and petty theft and serious offences – e.g. robbery and assault with a deadly weapon.  Violent crime – subscale of the general crime index which included 6 questions about violent interactions with others – e.g. gang fights, robbery and assault.  Arrest – official crime or arrest data.  Perpetration of intimate partner violence – with married, cohabiting and long-term dating partners (dating at least 6 months). | Race/ethnicity includes two dummy variables, African American and Hispanic, with non-Hispanic White as the omitted category, gender, chronic family poverty measures (the number of waves in poverty in the first four waves when adolescent participants were approximately 14 to 15.4 years old) – including income below the federal poverty line, unemployment, or receipt of public assistance, and any early adolescent general delinquency |
| Zych, 2021 | For the CSDD (The Cambridge Study in Delinquent Development) study, all participants were males born in London 1953. For the PYS (Pittsburgh Youth Study), all participants were boys in 1^st^, 4^th^ and 7^th^ grades in 1987-1988. For the z-proso (Zurich Project on the Social Development from Childhood to Adulthood) study, all participants had to be in their 1^st^ year of primary school in autumn 2004. | Not reported. | Gender and age (both controlled for through sample selection in CSDD and PYS only) |

| **Author** | **Selection (max = 6)** | | | | | | **Comparability (max = 2)** | | **Outcome (max = 5)** | | | | | **Total (max = 13)** |
| --- | --- | --- | --- | --- | --- | --- | --- | --- | --- | --- | --- | --- | --- | --- |
|  | Representa-tiveness of the exposed cohort (2) | | Selection of the non-exposed cohort (1) | Ascertainme-nt of exposure (1) | Adjustment made for baseline or prior offending (1) | Sample Size Justified and Satisfactory (1) | Controls for age and gender (0-1) and any other additional factor (0-1) either in selection of cohort or adjusted/stratified analysis (2) | | Assessment of offending outcome (1) | Was follow-up long enough for outcomes to occur? (1) | Follow-up complete, or unlikely to introduce bias (<20% lost or attrition described and accounted for in analysis) (2) | | Statistical test reporting clear, appropriate and complete (1) |  |
| Choi | 0 | 0 | 1 | 0 | 1 | 0 | 1 | 1 | 0 | 1 | 0 | 0 | 1 | 6 |
| Crosnoe | 0 | 1 | 1 | 0 | 1 | 0 | 1 | 1 | 0 | 1 | 0 | 1 | 0 | 7 |
| Defoe | 0 | 1 | 1 | 0 | 1 | 0 | 0 | 1 | 0 | 1 | 0 | 0 | 0 | 5 |
| Dodge | 0 | 0 | 1 | 1 | 1 | 0 | 0 | 1 | 0 | 1 | 0 | 0 | 0 | 5 |
| Farrington | 0 | 1 | 1 | 1 | 1 | 0 | 1 | 1 | 1 | 1 | 0 | 1 | 0 | 9 |
| Felson & Staff | 0 | 1 | 1 | 1 | 1 | 0 | 1 | 1 | 0 | 1 | 0 | 0 | 1 | 8 |
| Gremmen | 0 | 1 | 1 | 1 | 1 | 0 | 1 | 1 | 0 | 0 | 0 | 1 | 1 | 8 |
| Hemphill | 0 | 1 | 1 | 0 | 0 | 0 | 1 | 1 | 0 | 1 | 0 | 1 | 1 | 7 |
| Karriker-Jaffe | 1 | 1 | 1 | 1 | 1 | 0 | 1 | 1 | 1 | 1 | 0 | 0 | 1 | 10 |
| Lee | 0 | 1 | 1 | 0 | 1 | 0 | 0 | 1 | 0 | 1 | 0 | 1 | 1 | 7 |
| Lesner | 0 | 1 | 1 | 1 | 1 | 0 | 1 | 1 | 1 | 1 | 0 | 1 | 1 | 10 |
| Mercer | 0 | 0 | 1 | 0 | 1 | 0 | 0 | 1 | 1 | 1 | 0 | 1 | 0 | 6 |
| Sabates | 0 | 0 | 0 | 1 | 0 | 0 | 0 | 1 | 1 | 1 | 0 | 0 | 0 | 4 |
| Savage & Ellis | 0 | 1 | 1 | 0 | 0 | 0 | 1 | 1 | 0 | 1 | 0 | 1 | 1 | 7 |
| Savolainen | 0 | 0 | 1 | 0 | 1 | 0 | 1 | 1 | 1 | 1 | 0 | 0 | 1 | 7 |
| Smith | 0 | 1 | 1 | 1 | 1 | 0 | 0 | 1 | 1 | 1 | 0 | 1 | 0 | 8 |
| Zych | 0 | 1 | 1 | 1 | 0 | 0 | 0* | 0 | 1 | 1 | 0 | 1 | 0 | 6 |

*Note.* The total score for each study is scored between 0 – 13.

*Controls for age and gender in PYS and CSDD but not z-proso.
